# A Novel Human Intention Prediction Approach Based on Fuzzy Rules through Wearable Sensing in Human–Robot Handover

**DOI:** 10.3390/biomimetics8040358

**Published:** 2023-08-10

**Authors:** Rui Zou, Yubin Liu, Ying Li, Guoqing Chu, Jie Zhao, Hegao Cai

**Affiliations:** 1 State Key Laboratory of Robotics and Systems, Harbin 150001, China; zouruihit@163.com (R.Z.); 20b908054@stu.hit.edu.cn (G.C.); jzhao@hit.edu.cn (J.Z.); hgcai@hit.edu.cn (H.C.); 2 School of Management, Harbin University of Commerce, Harbin 150080, China

**Keywords:** human–robot handover, intention prediction, wearable sensing, robot control

## Abstract

With the use of collaborative robots in intelligent manufacturing, human–robot interaction has become more important in human–robot collaborations. Human–robot handover has a huge impact on human–robot interaction. For current research on human–robot handover, special attention is paid to robot path planning and motion control during the handover process; seldom is research focused on human handover intentions. However, enabling robots to predict human handover intentions is important for improving the efficiency of object handover. To enable robots to predict human handover intentions, a novel human handover intention prediction approach was proposed in this study. In the proposed approach, a wearable data glove and fuzzy rules are firstly used to achieve faster and accurate human handover intention sensing (HIS) and human handover intention prediction (HIP). This approach mainly includes human handover intention sensing (HIS) and human handover intention prediction (HIP). For human HIS, we employ wearable data gloves to sense human handover intention information. Compared with vision-based and physical contact-based sensing, wearable data glove-based sensing cannot be affected by visual occlusion and does not pose threats to human safety. For human HIP, we propose a fast handover intention prediction method based on fuzzy rules. Using this method, the robot can efficiently predict human handover intentions based on the sensing data obtained by the data glove. The experimental results demonstrate the advantages and efficacy of the proposed method in human intention prediction during human–robot handover.

## 1. Introduction

In recent years, human–robot collaboration has attracted considerable attention owing to its significant advantages [1,2,3]: on the one hand, robots can manage repetitive, simple, and cumbersome tasks; on the other hand, human workers can manage dexterous and complex tasks. Through human–robot collaboration, the efficiency of industrial manufacturing—especially dexterous and complex hybrid assembly tasks—can be significantly improved [4,5,6].

When human–robot collaboration is used for assembly tasks, object handover between humans and robots is basic and inevitable [7,8]. Through human–robot handover, considerable time and labor consumption can be saved in the assembly task; consequently, the assembly efficiency can be improved [9]. For example, in automobile assembly, for human workers, significant time and effort are required to collect and deliver parts to their colleagues (or obtain parts from their colleagues and put them in their proper places) during the assembly [10]. Human–robot handover can significantly reduce time cost and human effort consumption because robots are used as human colleagues to complete these simple but time-consuming operations [11].

To achieve natural and seamless object handover between a human and a robot, such as the natural and smooth object handover between a human and a human, coordination between a human and a robot in space and time is necessary [12,13]. To implement this coordination, the prediction of human handover intention is essential [14]. Human handover intention refers to what the human wants to do for the robot or what the human wants the robot to do in human–robot handover. Recently, related studies have been conducted on the prediction of human handover intention. The methods proposed in these studies can be divided into two categories: vision-based methods and physical contact-based methods.

For the vision-based methods, Rosenberger et al. used real-time robotic vision to grasp unseen objects and realized safe object-independent human-to-robot handover [15]. They used a Red-Green-Blue-Depth (RGBD) camera to obtain segmentation of a human body and hand segmentation to perform object detection. Therefore, they realized object-independent human-to-robot handover. Melchiorre et al. proposed a vision-based control architecture for human–robot handover applications [16]. They used a 3D-sensor setup to predict the position of a human worker’s hand and adapted the pose of the tool center point of the robot to the pose of the hand of the human worker to realize a human–robot handover. Ye et al. used a vision-based system to construct a benchmark for visual human-human object handover analysis [17]. They used Intel Realsense cameras to record RGBD videos of the two humans’ handover process, and these videos could be used to predict the human’s handover intention to improve the efficiency of human–robot handover. Wei et al. proposed a vision-based approach to measure environmental effects on the prediction of human intention [18]. They used their approach in human–robot interaction and realized safe, efficient human–robot interaction. Liu et al. used a Kinect V2 camera to collect a human–robot interaction dataset [19]. Based on the dataset, they used the Spatial-Temporal Graph Convolutional Network with Long Short Term Memory (ST-GCN-LSTM) layers model and the YOLO model to recognize humans’ intentions during human–robot handover. Chan et al. used a vision-based system to detect humans’ behaviors [20]. According to the detection result, they computed object orientations by an affordance and distance minimization-based method to realize smooth human–robot handover. Yang et al. proposed a vision-based system to enable unknown objects to be transferred between a human and a robot [21]. They employed the Azure Kinect RGBD camera to capture RGBD images of a human’s hand and predict the human’s handover intention, thereby realizing handing over diverse objects with arbitrary appearance, size, shape, and deformability.

However, these vision-based methods will not work in the case of visual occlusion. In addition, the vision system can only capture the motion of humans in a specific area, which considerably limits the range of humans’ work activities.

For the physical contact-based methods, Alevizos et al. used force/torque sensors to measure the interaction force/torque between a human and a robot [22]. Therefore, they proposed a method to predict a human’s motion intention to realize a smooth, intuitive physical interaction with the human. Wang et al. built a tactile information dataset for physical human–robot interaction [23]. They used a tactile sensor to record 12 types of common tactile actions of 50 subjects. Using this dataset, human intentions could be predicted during physical human–robot interaction. Chen et al. [24] proposed a systematic approach to adjust admittance parameters according to human intentions. They used a force/torque sensor installed at the endpoint of the manipulator to measure interaction force, which was used to predict human intentions. Yu et al. proposed a novel approach to estimate human impedance and motion intention for constrained human–robot interaction [25]. They used angle and torque sensors in the joint to measure interaction force and motion information to predict human intentions to complete natural human–robot interaction. Li et al. proposed a control scheme for physical human–robot interaction coupled with an environment of unknown stiffness [26]. During the physical human–robot interaction in their work, the human force was measured by a 6-DOF T/F sensor, Nano 25 (ATI Industrial Automation Inc., Apex, NC, USA) at the end-effector, and the human intention was estimated based on the measured human force. Hamad et al. proposed a robotic admittance controller which was used to adaptively adjust its contribution to a collaborative task performed with a human worker [27]. In their work, a force sensor (Mini40, ATI Inc., Columbia, MD, USA) was used to measure the force applied by the human worker. The human worker’s intention was predicted using the measured interaction force. Khoramshahi et al. designed a dynamical system method for detection and reaction to human guidance during physical human–robot interaction [28]. They proposed a human intention detection algorithm to predict the human’s intention using the human force measured by the force/torque sensor (Robotiq FT300, Robotqi, Quebec City, Canada) mounted on the end-effector. Li et al. developed an assimilation control approach to reshape the physical interaction trajectory in the interaction task [29]. They estimated the human’s virtual target from the interaction force measured by a six-axis force sensor (SRI. M3703 C, Sunrise Instruments, Canton, MI, USA), and by employing this method, the robot could adapt its behavior according to the human’s intention.

However, these physical contact-based methods depend on physical contact between the human and the robot in the process of human–robot handover, which may endanger humans.

To overcome the shortcomings of the methods based on vision and physical contact, recently, wearable sensing technology has been applied in human–robot handover. In [30], Zhang et al. developed an electromyography (EMG) signal-based human–robot collaboration system. In the system, they used EMG sensors to sample EMG signals of a human’s forearm. Based on the sampled EMG signals, they developed an algorithm to control a robot to complete several motions. In [31], Sirintuna et al. used neural networks trained by EMG signals to guide the robot to perform specific tasks in physical human–robot interaction. The EMG signals were sampled by four wearable EMG sensors on various muscles of the human arm. In [32], Mendes et al. proposed a deep learning-based approach for human–robot collaboration. In their method, surface EMG signals were sampled by wearable EMG sensors to control a robot’s mobility in human–robot interaction. In [33], Cifuentes et al. developed a wearable mobile robot’s controller to track the motion of a human in human–robot handover. In this controller, an inertial measuring unit (IMU) that can be worn and laser rangefinder were used to sample human motion data, which were fused to build a human–robot handover strategy. In [34], Artemiadis et al. used wearable EMG sensors to detect signals from human upper limb muscles and and created a user-robot control interface. In [35], wearable IMU and EMG sensors were incorporated into the robot’s sensing system to enable independent control of the robot. In [14], Wang et al. used wearable EMG and IMU sensors to collect motion data of the human forearm and hand. These motion data were used to predict human handover intentions. Therefore, to the best of our knowledge, this was the only research work in which wearable sensing technology was used to predict human handover intentions.

However, the above research work has three disadvantages: (1) These wearable sensing-based methods mainly aim at controlling the low-level motion of the robot. Limited research addressing human HIP has been conducted. (2) Current research which uses wearable sensing to predict human handover intentions adopt EMG sensors. However, the signals of EMG sensors are easily disturbed by physiological electrical signals, which will reduce the prediction accuracy of human handover intention. (3) Current research which uses wearable sensing to predict human handover intentions can only collect human motion signals from the forearm. However, human motion signals from other body parts, such as five fingers, cannot be collected; thus, human intentions represented by other body parts (such as human intentions represented by gestures) cannot be predicted.

To overcome the above three shortcomings, this study proposes a new method to predict human handover intentions in human–robot collaboration. In our methods, firstly, we employ a wearable data glove to obtain human gesture information. Thereafter, we propose a fast human HIP model based on fuzzy rules. Finally, human gesture information is input to the human HIP model to obtain the predicted human handover intention. Through our method, the robot can predict human handover intentions accurately and collaborate with a human to finish object handover efficiently. The following are the study’s primary contributions.

- We developed a novel solution using wearable data glove to predict human handover intentions in human–robot handover.
- We used a data glove instead of EMG sensors to obtain human handover intention information, which avoids the disadvantage that EMG sensors’ signals are easily disturbed by physiological electrical signals. This improved the prediction accuracy of human handover intentions.
- We collected human gesture information to predict human handover intention, which enabled the robot to predict different human handover intentions.
- We proposed a fast HIP method based on fuzzy rules. The fuzzy rules were built based on thresholds of bending angles of five fingers rather than the thresholds of original quaternions of IMUs on five fingers. Using our method, the prediction of human handover intentions could be faster.
- We conducted experiments to verify the effectiveness and accuracy of our method. The experimental results showed that our method was effective and accurate in human HIP.

In what follows, the overall framework of human–robot handover is described in Section 2.1. The human HIS method is described in Section 2.2. The human HIP method is given in Section 2.3. We carry out a series of experiments to testify and evaluate the efficiency and accuracy of the proposed approach in Section 3. The conclusions of the research findings of this study are provided in Section 4.

## 2. Materials and Methods

### 2.1. Overall Framework of Human–Robot Handover

**Figure 1:**
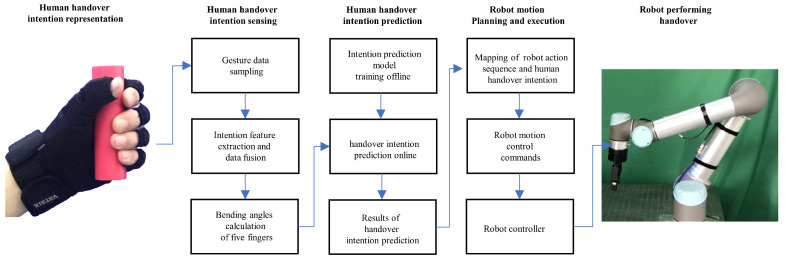
Overall framework of human–robot handover.

As shown in Figure 1, the overall framework of human–robot handover consists of three modules: the human HIS module, the human HIP module, and the robot motion planning and execution module.

To deliver an object to a human (or receive an object from a human) seamlessly and naturally during handover, the robot must predict human handover intentions. Before the prediction of human handover intention, human handover intention information needs to be collected. The collection of human handover intention information is conducted in the human HIS module. In this module, human handover intention information is represented by human gestures (the shape of five fingers). Consequently, the collection of human handover intention information is realized by collecting data of human gestures. The data collection of human gestures is conducted by a wearable data glove. The wearable data glove contains six IMUs. Each IMU includes a gyroscope, accelerometer, and magnetometer incorporated with a proprietary adaptive sensor fusion algorithm to provide original gesture data. The original gesture data are processed by data fusion and feature extraction. Subsequently, the bending angle of each finger is calculated and then sent to the human HIP module through a wireless device to predict human handover intentions.

The human HIP module consists of an offline human HIP model training unit and an online human HIP unit. In the offline model training unit, an offline dataset, which is composed of gesture data and corresponding intention labels, is used to construct a group of fuzzy rules. The fuzzy rules are used to generate a human HIP model. This generated model is used by the online human handover intention prediction unit. In the online human HIP unit, the bending angles of five fingers from the human HIS module are used by the human HIP model as input data. According to the input data, the human HIP model calculates the prediction result of human handover intention. The prediction result of human handover intention is input into the robot motion planning and execution module. According to the prediction result of human handover intention, the robot motion planning and execution module generate corresponding action sequences and motion control instructions, which control the robot to perform a specific movement in a human–robot handover.

The robot motion planning and execution module is composed of a motion planning unit and an execution unit. In the motion planning unit, we generate an action sequence set by human teaching and robot learning. In the action sequence set, each action sequence corresponds to a specific human handover intention, which forms a map between action sequences and human handover intentions. The mapping of action sequences and human handover intentions is queried by the robot when the human HIP result is calculated. The queried action sequence corresponding to the human HIP result is activated. Motion control instructions corresponding to the activated action sequence are subsequently generated and then sent to the execution unit to control the motion of the robot to complete the whole handover process.

Because this study focuses on natural wearable sensing and human HIP, in the following sections, we will describe the human HIS module and the human HIP module.

### 2.2. Human Handover Intention Sensing

**Figure 2:**
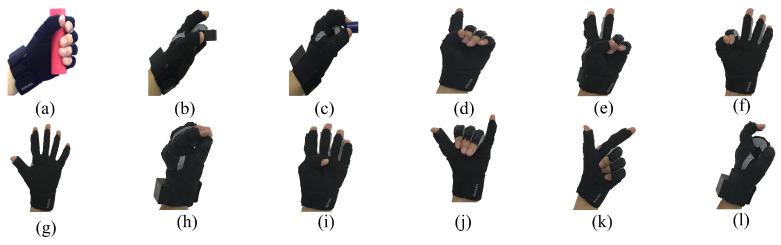
Human handover intentions are represented by wearing the data glove: (**a**–**c**) Give. (**d**–**f**) Need. (**g**) Rotate arm. (**h**) Rotate hand. (**i**) Move up. (**j**) Move down. (**k**) Move close. (**l**) Move far.

In this study, to verify the effectiveness and advantages of our proposed method, we selected 12 human gestures to represent human handover intentions as shown in Figure 2. The reason why we selected these 12 human gestures is that: We ask 5 individuals (two females and three males) to conduct handover tasks by delivering/picking up objects to/from a robot. By investigating and studying their handover manners, we find that the selected 12 kinds of handover intentions are commonly used for their handover intention expression in the human–robot handover process. This is why we selected these 12 gestures. These intentions include (a) Give the big red cylinder, (b) Give the middle black cylinder, (c) Give the small blue cylinder, (d) Need the big red cylinder, (e) Need the middle black cylinder, (f) Need the small blue cylinder, (g) Rotate arm, (h) Rotate hand, (i) Move up, (j) Move down, (k) Move close, and (l) Move far.

We employed human gestures to represent human handover intentions because they are more advantageous compared to other approaches [36]. For example, compared with using natural language to represent human handover intentions [37], using human gestures to represent human handover intentions avoids interference from noise in the industrial environment. Compared with using the human gaze to represent human handover intentions [38,39], using human gestures to represent human handover intentions will be more natural and intuitive.

During human–robot collaboration, such as in assembly according to assembly requirements, the human represents the corresponding handover intention by specific gestures to ask the robot to offer assistance. To efficiently assist humans, the robot must understand the handover intention represented by the human. Previously, the robot needs to obtain corresponding human gesture information.

**Figure 3:**
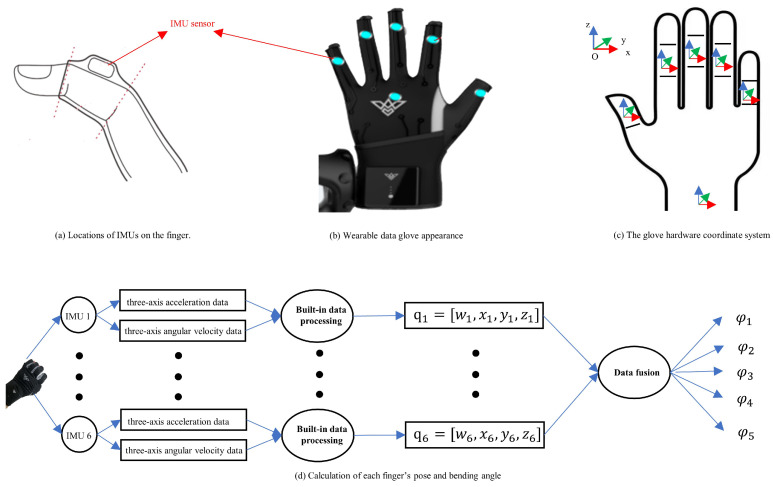
Wearable data glove.

Human gesture information corresponding to human handover intention is obtained by a wearable data glove as shown in Figure 3a,b. The wearable data glove can be easily worn on a human’s hand. The wearable data glove contains six six-axis IMUs [40,41,42]. One of the IMUs is located on the back of the hand, and the other five IMUs are respectively located on the second section of each finger. The sensing data of these IMUs can be sent out through wireless communication (Bluetooth) in the data glove [43,44].

Human gestures can be expressed through the attitudes of five fingers and the back of the hand as shown in Figure 3d. These attitudes can be sensed by the six IMUs in the data glove. The sensing data obtained by each IMU include the original three-axis acceleration data as well as the original three-axis angular velocity data [45]. These original data can be transformed to quaternion by the built-in data processing algorithm in an IMU [46]. The quaternion obtained from each IMU can be expressed as

(1) $$q={\left[w,x,y,z\right]}^{T}$$

where w,x,y,z are components of the quaternion. The quaternion is calculated according to an established world coordinate system. The IMU’s sampling rate in the data glove is 120 Hz.

All the quaternions obtained by the six IMUs can be fused to quantitatively describe human gestures as

(2) $$q=[q_{1},\cdots ,q_{i},\cdots ,q_{n}]$$

where $q_{i}$ represents the quaternion obtained by the $i^{th}$ IMU. *q* can be used to quantitatively describe human gestures. Furthermore, *q* is input to the human HIP module to calculate the predicted result of human handover intention, which is described in detail in the following section.

### 2.3. Human Handover Intention Prediction

To predict human handover intention, in this section, we propose a fast human HIP method based on fuzzy rules [47,48]. First, we employ a statistical method to obtain thresholds of bending angles of five fingers for each human handover intention. Thereafter, based on the obtained thresholds of bending angles of five fingers, a collection of fuzzy rules are defined to predict human handover intention.

We request that the experimenters present different handover intentions based on the specified gesture expressions as shown in Figure 2 and wearing the wearable data glove. We collected the handover intention data with the data glove within our experimental platform as shown in Section 2.4. To differentiate between different human handover intentions, a traversal procedure compares each element to the others in the collected handover intention data to obtain the minimum and maximum values of every element. In particular, we can obtain the lower and upper thresholds for each collected handover intention dataset. Because 12 types of human handover intentions are considered in this study, we can correspondingly obtain 12 groups of upper and lower thresholds. If we obtain one set of handover intention data, by judging which group of upper and lower thresholds the data are within, we can recognize the handover intention represented by the data.

As mentioned in Equation (2), the handover intention data are represented by quaternions of the six IMUs. There are 4 elements (q.x,q.y,q.z,q.w) for each IMU, and then there are 24 elements for the six IMUs. The calculation load to determine the upper and lower thresholds for the 24 elements is heavy. Therefore, to reduce the load of calculation, we propose a new method to represent human handover intention. Generally, when we grasp an object with a different gesture, the bending angles of the five fingers are different. Therefore, the bending angles of five fingers can be used to represent the human handover intention. To calculate the bending angles of five fingers, we need to understand the glove hardware coordinate system. The glove hardware coordinate system is defined by the left-hand coordinate system as shown in Figure 3c. In Figure 3c, the red coordinate axis is the X-axis, the green coordinate axis is the Y-axis, and the blue coordinate axis is the Z-axis. The bending angle of each finger can be calculated as follows.

First, we calculate the angular displacement of the intermediate section of the finger relative to the wrist joint as follows [49]

(3) $$\Delta_{q}=q_{fm}^{-1}q_{w}$$

where $\Delta_{q}=\left[\Delta_{q0},\Delta_{q1},\Delta_{q2},\Delta_{q3}\right]$ represents the angular displacement of the intermediate section of the finger relative to the wrist, $q_{fm}=\left[q_{fm.w},q_{fm.x},q_{fm.y},q_{fm.z}\right]$ represents the quaternion of the intermediate section of the finger, and $q_{w}=\left[q_{w.w},q_{w.x},q_{w.y},q_{w.z}\right]$ represents the quaternion of the wrist.

Second, in general, object rotations can be represented in several ways: a direction cosine matrix or a projection angle or an Euler decomposition or other representations. The reason why we select an Euler decomposition is that the Euler angle is easy for us to understand and use, and it is also a quite memory-efficient representation compared with other representations.Therefore, for convenience, we transfer $\Delta_{q}$ into the Euler angle as [50]

(4) $$\left[\begin{array}{c} \varphi \\ \theta \\ \phi \end{array}\right]=\left[\begin{array}{c} atan2(2\left(\Delta_{q0}\Delta_{q1}+\Delta_{q2}\Delta_{q3}\right),1-2\left(\Delta_{q1}^{2}+\Delta_{q2}^{2}\right))\\ asin(2\left(\Delta_{q0}\Delta_{q2}-\Delta_{q3}\Delta_{q1}\right))\\ atan2(2\left(\Delta_{q0}\Delta_{q3}+\Delta_{q1}\Delta_{q2}\right),1-2\left(\Delta_{q2}^{2}+\Delta_{q3}^{2}\right)) \end{array}\right]$$

where φ,θ,ϕ are the rotation angles around the X-axis, Y-axis, and Z-axis, respectively. Because the finger only bends around one axis (in this paper, the axis is the X axis); therefore, ϕ and θ are equal to 0. In particular, φ can be used to represent the bending angle of each finger.

Finally, the gesture can be represented as

(5) $$\varphi =[\varphi_{1},\varphi_{2},\varphi_{3},\varphi_{4},\varphi_{5}]$$

where $\varphi_{1},\varphi_{2},\varphi_{3},\varphi_{4},\varphi_{5}$ represent the bending angles of the thumb, index finger, middle finger, ring finger, and pinky finger, respectively. φ represents the bending angles of five fingers, which can be used to describe gestures. The process of calculating bending angles of the five fingers is shown in Figure 3d.

Therefore, to predict the intention, we only need to identify the lower and upper thresholds of the bending angles of five fingers. This will obviously reduce loads of calculations.

In Table 1, for the human handover intentions considered in this paper, we extract 12 sets of thresholds. The corresponding method is described in Section 3.2.

**Figure 4:**
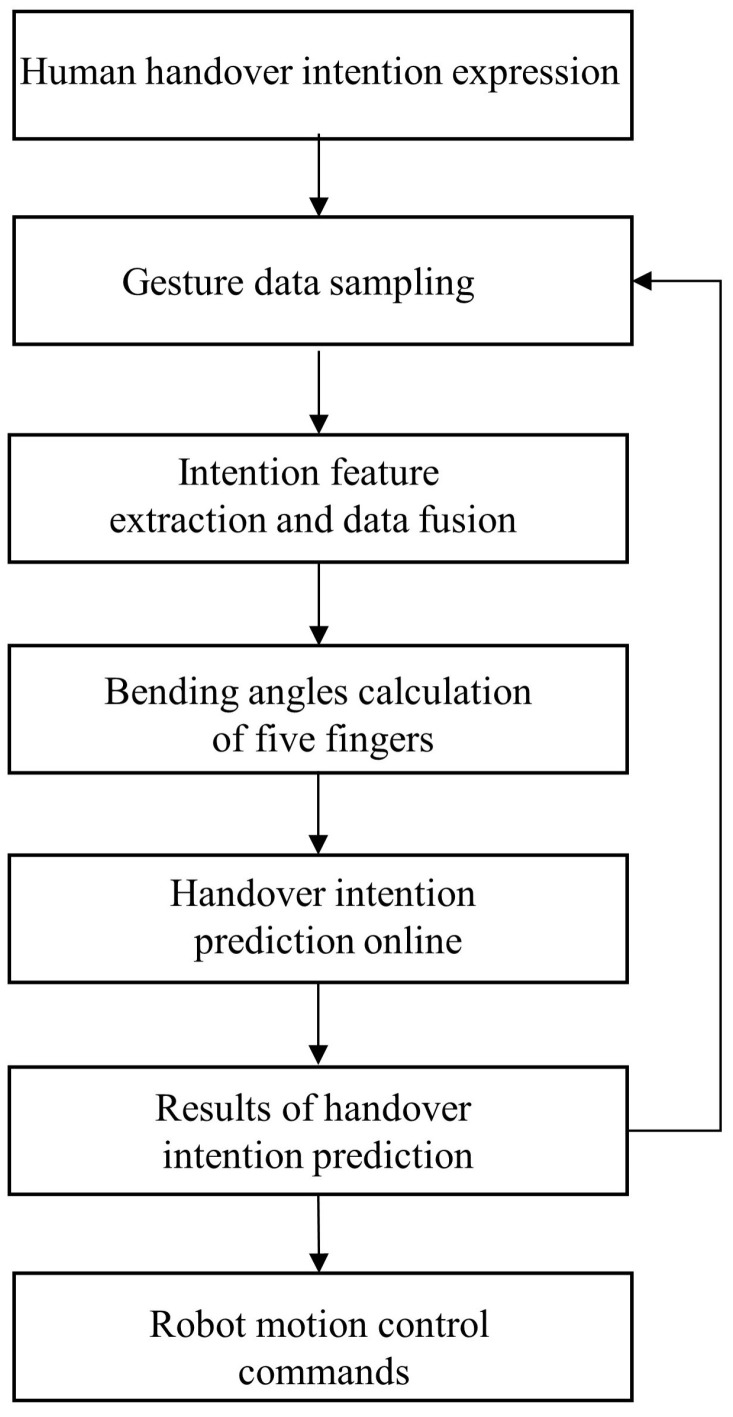
The generalized flowchart of the proposed approach.

Based on these thresholds, a collection of fuzzy rules are defined to recognize human handover intentions expressed by an individual [51,52]. The fuzzy rules can be depicted by

(6) $$\begin{array}{cc} \; & \mathrm{If}\;\varphi_{1}\in \left[L_{\varphi_{1},i},H_{\varphi_{1},i}\right]\;\mathrm{and}\;\varphi_{2}\in \left[L_{\varphi_{2},i},H_{\varphi_{2},i}\right]\;\mathrm{and}\;\varphi_{3}\in \left[L_{\varphi_{3},i},H_{\varphi_{3},i}\right]\;\mathrm{and}\;\varphi_{4}\in \left[L_{\varphi_{4},i},H_{\varphi_{4},i}\right]\;\mathrm{and}\\ \; & \varphi_{5}\in \left[L_{\varphi_{5},i},H_{\varphi_{5},i}\right],\\ \; & \mathrm{Human}\;\mathrm{intention}\to I_{i} \end{array}$$

where *H* and *L* stand for high and low thresholds, respectively, and $I_{i}$ stands for a human handover intention. The fuzzy variables are $\varphi_{1},\varphi_{2},\varphi_{3},\varphi_{4},\varphi_{5}$. Therefore, if the bending angle of the thumb $\varphi_{1}$ is within the bounds of $\left[L_{\varphi_{1},i},H_{\varphi_{1},i}\right]$, the bending angle of the index finger $\varphi_{2}$ is within the bounds of $\left[L_{\varphi_{2},i},H_{\varphi_{2},i}\right]$, the bending angle of the middle finger $\varphi_{3}$ is within the bounds of $\left[L_{\varphi_{3},i},H_{\varphi_{3},i}\right]$, the bending angle of the ring finger $\varphi_{4}$ is within the bounds of $\left[L_{\varphi_{4},i},H_{\varphi_{4},i}\right]$, and the bending angle of the small finger $\varphi_{5}$ is within the bounds of $\left[L_{\varphi_{5},i},H_{\varphi_{5},i}\right]$, we can conclude that the human desires to carry out the handover intention $I_{i}$. These thresholds are determined according to information gathered from user studies. The experiment section includes a presentation of the results. The generalized flowchart of the proposed approach is shown in Figure 4.

### 2.4. Experimental Setup

**Figure 5:**
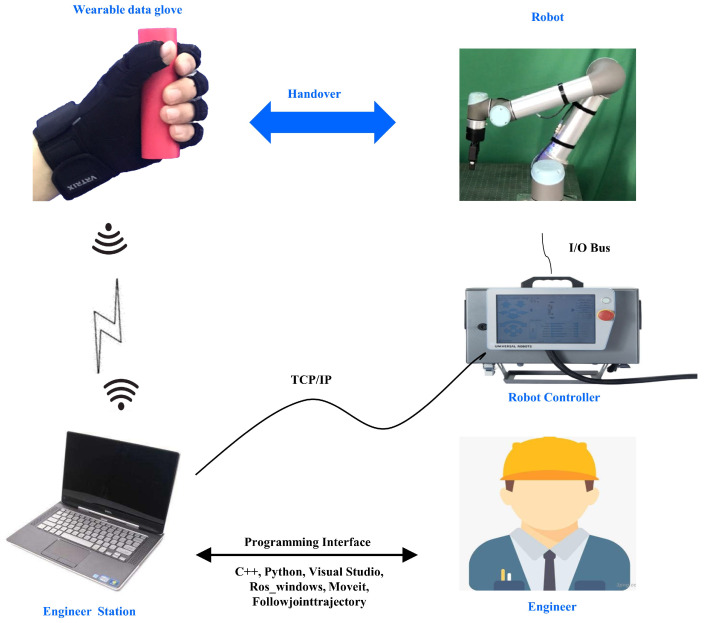
Experimental platform.

The experimental platform consists of a wearable data glove, a UR5 robot, a controller, and an engineer station. The components of the experimental platform communicate through Bluetooth and transmission control protocol/Internet Protocol (TCP/IP). During human–robot handover, a data glove is worn on the left hand of a human. The data glove contains six IMUs located on five fingers and the back of the hand. These IMUs send the sensing data of human handover intentions to the engineer station in real time through Bluetooth. The engineer station receives and processes the sensing data from the wearable data glove and runs a human HIP algorithm to obtain the result of human handover intention prediction. The data receiving, data processing, and human HIP algorithm are programmed by the engineer using C++ and Python through programming interfaces such as visual studio 2017 and ROS. Moveit is the most widely used software for robot motion planning. The Followjointtrajectory action is a ROS node that provides an action interface for tracking trajectory execution. It passes trajectory goals to the robot controller, and reports success when they have finished executing. In this paper, we will use Moveit and the Followjointtrajectory action node for robot motion planning.The experimental platform is shown in Figure 5. Our experiments include 12 human handover intentions as shown in Figure 2. These human handover intentions are expressed by the experimental participant wearing the data glove on the left hand.

In our experiment, we first conduct a test to evaluate if the information of human handover intentions can be successfully sensed by the wearable data glove. Secondly, we describe how to construct the prediction model based on the sensed information and evaluate the performance of the prediction model. Finally, we conduct online human–robot handover experiments to verify whether the robot can correctly predict human handover intentions and collaborate with the experimental participant to complete the whole handover process.

## 3. Results and Discussion

### 3.1. Sensing of Human Handover Intentions

**Figure 6:**
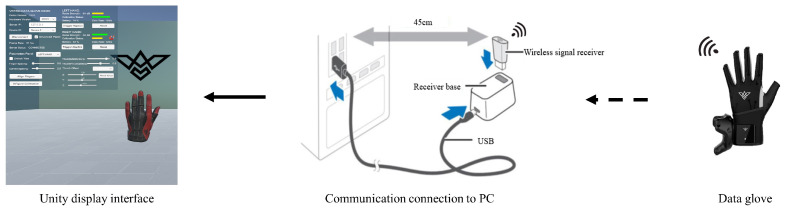
Communication connection between data glove, PC, and Unity.

In this section, we experiment to evaluate the effectiveness of the proposed approach in terms of the sensing ability of human intentions. During the experiment, a human wearing a data glove performs several intentions with different gestures. The IMUs in the data glove sample pose information of the human’s fingers and send the sampled data to Unity on PC as shown in Figure 6. After Unity has received the sampled data, it restructures and displays human gestures in the virtual environment.

**Figure 7:**
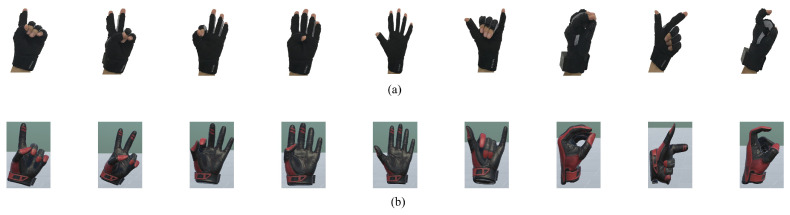
Sensing of human gestures. (**a**) Human expresses the numbers 1–9 by wearing a data glove with different gestures. (**b**) Unity reconstructs and displays human gestures using the sensing data in the virtual reality environment.

**Figure 8:**
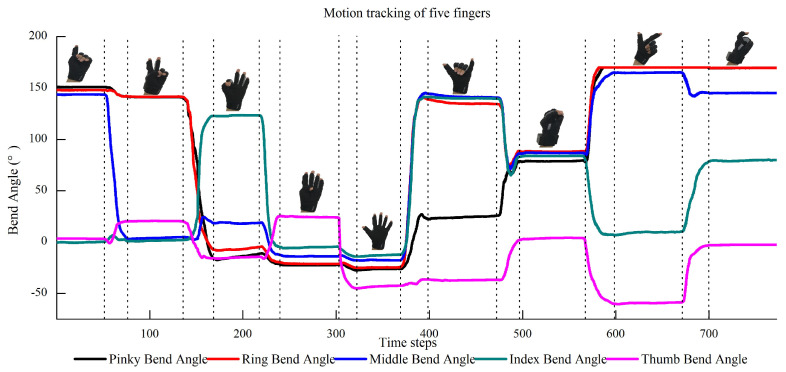
Real-time tracking of the motion of five fingers when the human performs intentions by different gestures.

Figure 7 shows the sensing results of humans’ intentions. It can be observed that when the human wearing a data glove performs different intentions of “One”, “Two”, “Three”, “Four”, “Five”, “Six”, “Seven”, “Eight”, and “Nine” by different gestures, the data of these gestures can be sampled by the data glove and sent to Unity. Unity successfully restructured and displayed these gestures in the virtual environment as shown in Figure 7. Furthermore, we record the bending angles of the five fingers when the human performs intentions by different gestures as shown in Figure 8. It can be observed that the motion of five fingers can be tracked successfully in real time. Therefore, human intentions can be successfully sensed by our method. The sensing data are used to predict human handover intentions.

### 3.2. Prediction Model Construction

**Figure 9:**
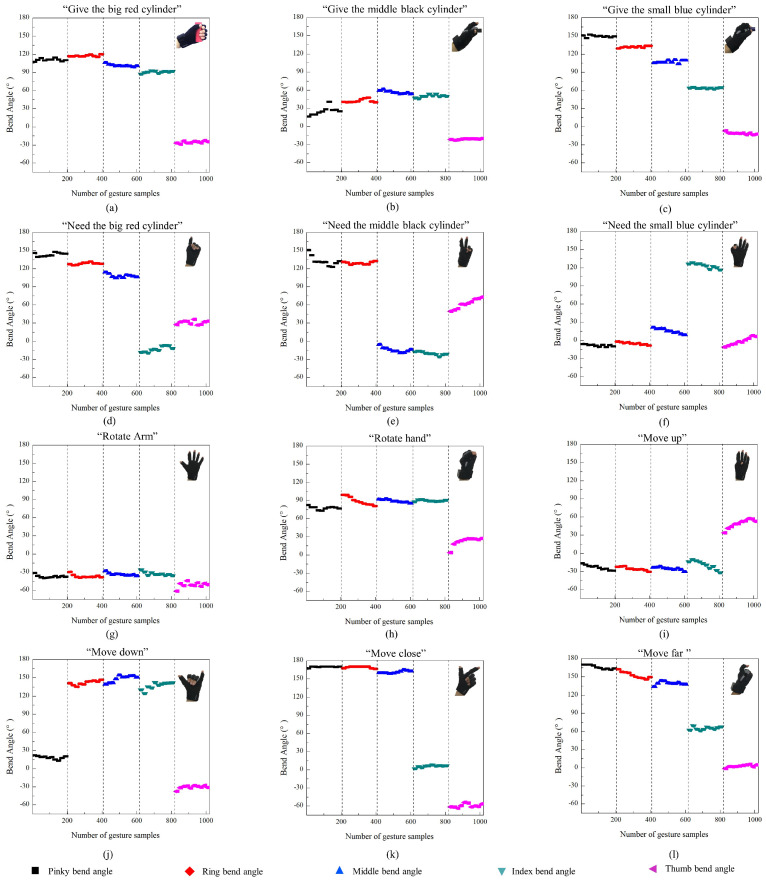
Bending angles of five fingers for all the 12 handover intentions in the 200 presentations.

We asked the experimental participant wearing the wearable data glove to present 12 different handover intentions as depicted in Figure 2. Each handover intention is presented 200 times. For each presentation, the pose data of five fingers are sampled by the IMUs in the data glove. Using these sampled data, we can calculate the bending angles of five fingers using Equations (3)–(5). The bending angles of five fingers for all the 12 handover intentions in the 200 presentations are shown in Figure 9.

The human HIP model is constructed by establishing the upper and lower thresholds of the bending angles of five fingers for each human handover intention. By employing the data sampled from 200 presentations as shown in Figure 9, we can obtain the maximum and minimum values of the bending angles. Thereafter, the upper and lower thresholds of the bending angles can be established by the maximum and minimum values. Because 12 types of human handover intentions are considered in this study, we can obtain 12 groups of upper and lower thresholds correspondingly as shown in Table 1. If we obtain one set of handover intention data, by judging which group of upper and lower thresholds the data are within, we can predict the handover intention represented by the data.

### 3.3. Performance Evaluations of the Prediction Model

We evaluate these 12 human handover intentions’ prediction accuracy using a variety of techniques. These techniques comprise our method, linear discriminant analysis (LDA) [53], support vector machine (SVM) [54], and k-nearest neighbor (KNN) [55]. An identical operation station is used to test each algorithm. In order to evaluate the performance of the proposed model, we ask five subjects (two females and three males) within an age range of 25–33 to collaborate with the robot to conduct handover tasks using the proposed approach. The prediction accuracy evaluation uses 6000 sets (500 sets per human handover intention) of human handover intention features. The k-fold cross-validation method is utilized to unbiasedly check and evaluate these approaches’ generalization in human HIP [56]. We divided the collected data into 10 equally sized portions according on the empirical evidence [57]. For the sake of testing the suggested prediction algorithm, one of the ten subsets is utilized as the validation (prediction) set, while the other nine are combined to produce a training set. As a consequence, the cross-validation process is performed 10 times, and each subset is used as the validation data once. As shown in Table 2, for each human intention, the accuracy estimate is calculated by averaging the nine prediction outcomes. It can be concluded that the proposed method can successfully predict all the human handover intentions with a higher accuracy than other approaches.

**Figure 10:**
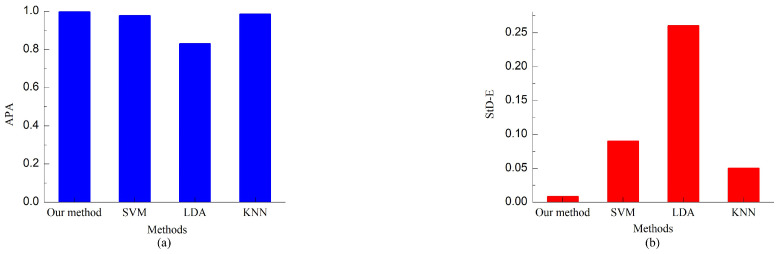
Comparison results of different methods in human handover intention prediction. (**a**) APA. (**b**) StD-E.

The results of average prediction accuracy (APA) of the 12 handover intentions are illustrated in Figure 10a, and the standard deviation of the prediction errors (StD-E) of the 12 handover intentions are illustrated in Figure 10b. The histogram shows that the APA results of these methods are 99.6%, 97.6%, 82.9%, and 98.4%, respectively. It can be concluded that the proposed approach can predict all human handover intentions in human–robot collaboration more accurately than other methods. Additionally, Figure 10 demonstrates that the StD-E produced by the proposed model is approximately 0.008, which is significantly less than the StD-Es of SVM (0.09), LDA (0.26), and KNN(0.05). Consequently, it can be stated that our method is more reliable in predicting human handover intentions.

### 3.4. Online Human–Robot Handover

In this section, we conduct an online human-to-robot object handover experiment. During the handover process, the experimental participant wears a data glove on the left hand, picks up a cylinder, and expresses the handover intention to the robot—“Give the cylinder to you”. The sensing data of the human handover intention are sent to the robot through wireless communication. Using the received sensing data, the robot predicts the handover intention of the human. After the human intention is successfully predicted, the robot receives the cylinder from the human hand and places the cylinder in the correct place.

**Figure 11:**
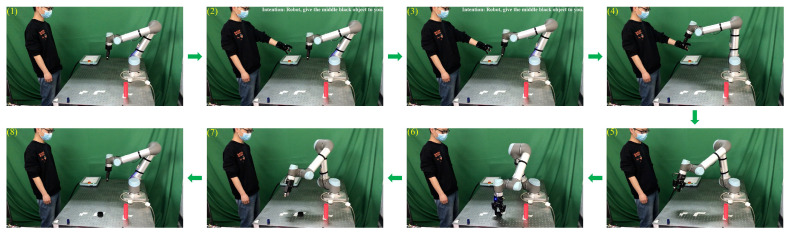
Process of human-to-robot handover.

Figure 11 shows the process of object handover from the human to the robot. As shown in Figure 11((1),(2)), the experimental participant grasps the middle black cylinder randomly and begins to hand it over to the robot. After the robot successfully predicts the human’s handover intention, it picks up the delivered cylinder from the human’s hand, as shown in Figure 11((3)–(5)). Subsequently, the robot places the cylinder in the correct position shown in Figure 11(6) and returns to the initial position, as shown in Figure 11((7),(8)). These pictures taken during the experiment show that the robot successfully predicts the human handover intention through the proposed approach and further collaborates with the human to complete the whole handover process.

### 3.5. Robot Motion Mode Adjustment

**Figure 12:**
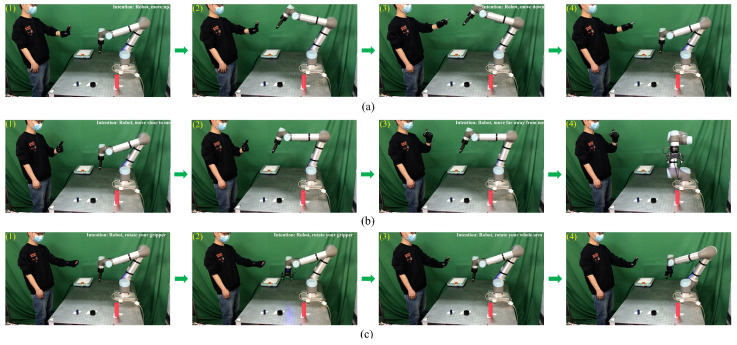
Robot motion mode adjustment according to the human intentions. (**a**) Move up and down. (**b**) Move close and far away. (**c**) Rotate the gripper and the arm.

In this section, we conduct an online robot motion mode adjustment experiment. During the handover process, the experimental participant wears a data glove on the left hand and expresses the “Move up”, “Move down”, ”Move close”, “Move far away”, “Rotate the gripper” and ”Rotate the arm” intentions to the robot separately. The robot predicts the intentions of the experimental participant and adjusts its motion mode according to the prediction result. Figure 12 shows the process of the robot motion mode adjustment using our approach. As shown in Figure 12a((1),(2)), once the human expresses the “Move up” intention, the robot moves its gripper upward. When the human expresses the “Move down” intention, the robot moves its gripper down as shown in Figure 12a((3),(4)). After the human presents the “Move close” intention, the robot moved closer to the human as shown in Figure 12b((1),(2)). After the human presents the “Move far away” intention, the robot moved far away from the human as shown in Figure 12b((3),(4)). From Figure 12c((1),(2)), we can observe that after the human expresses the “Rotate your gripper” intention, the robot rotated its gripper. From Figure 12c((3),(4)), we can observe that after the human expresses the “Rotate your arm” intention, the robot rotated its arm. These pictures taken during the experiment show that through the proposed approach, the robot successfully predicts the human’s intentions and adjusts its motion mode correspondingly.

### 3.6. Online Robot-Human Handover

In this section, we conduct an online robot-to-human object handover experiment. During the handover process, the experimental participant wears a data glove on the left hand and expresses the handover intention to the robot using the gesture “I need the big red cylinder from you”. The sensing data of the human handover intention are sent to the robot through wireless communication. Using the received sensing data, the robot predicts the handover intention of the human. After the human handover intention is successfully predicted, the robot correctly grasps the object and hands it over to the experimental participant.

**Figure 13:**
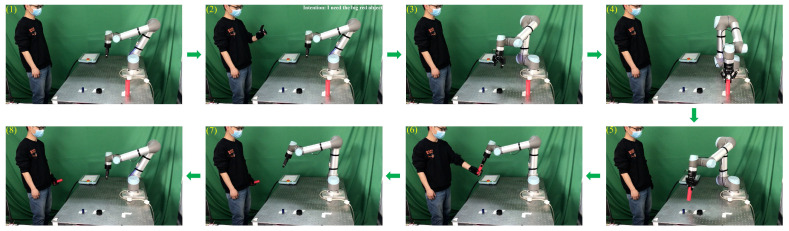
The process of robot-to-human handover.

Figure 13 shows the process of object handover from the robot to the human. As shown in Figure 13((1),(2)), the experimental participant expresses his intention to the robot—“I need the big red cylinder from you”. The robot receives sensing data from the data glove and predicts the handover intention of the experimental participant. Once the robot successfully predicts the handover intention, it grasps the big red cylinder and hands it over to the experimental participant, as shown in Figure 13((3)–(6)). After the experimental participant receives the desired cylinder from the robot, the robot moves back to its starting position, which is shown in Figure 13((7),(8)). These pictures taken during the experiment show that the robot successfully predicts the human’s handover intention through the proposed approach and further collaborates with the human to complete the whole handover process.

## 4. Conclusions

A novel human HIP approach has been proposed in this study to allow robots to predict human handover intentions. This proposed approach includes human HIS and human handover prediction.

- For human HIS, a wearable data glove is used to sense human handover intention information. Compared with vision-based and physical contact-based sensing methods, data glove-based sensing is not affected by visual occlusion; thus, it does not pose threats to human safety.
- For human HIP, a fast HIP method based on fuzzy rules has been proposed. Using this method, the robot can efficiently predict human handover intentions based on the sensing data obtained by the data glove.

The experimental results show the benefits and efficiency of the proposed method in human HIP during human–robot collaboration.

The proposed method still has several limitations, which motivate our future research. First, the prediction accuracy of the proposed method is still not 100%. To improve the performance, in future, we will consider developing human handover intention prediction method based on multimodal information. The multimodal information may be obtained by combining wearable sensing with human gaze, speech, etc. Second, we mainly address human handover intention sensing and prediction in this paper. In future, we plan to integrate the proposed method with motion planning to solve the human–robot handover problem.

## Figures and Tables

**Table 1:** Thresholds for each human handover intention.

Intentions	$\varphi_{1}/{(}^{\circ })$	$\varphi_{2}/{(}^{\circ })$	$\varphi_{3}/{(}^{\circ })$	$\varphi_{4}/{(}^{\circ })$	$\varphi_{5}/{(}^{\circ })$
Give the big red cylinder	{−29, −21}	{87, 94}	{98, 106}	{115,120}	{107,115}
Give the middle black cylinder	{−23,−19}	{46,55}	{53,62}	{39,48}	{16,42}
Give the small blue cylinder	{−14,−6}	{62,66}	{103,111}	{129,134}	{146, 153}
Need the big red cylinder	{26,37}	{−19,−5}	{104,115}	{125,132}	{139,149}
Need the middle black cylinder	{49,73}	{−25,−15}	{−20,−5}	{126,133}	{122,151}
Need the small blue cylinder	{−11,8}	{117,130}	{8,22}	{−9,−1}	{−12,−5}
Rotate arm	{−62,−44}	{−36,−25}	{−37,−27}	{−39,−29}	{−40,−31}
Rotate hand	{4,28}	{88,93}	{85,93}	{80,100}	{72,83}
Move up	{33,58}	{−32,−9}	{−31,−21}	{−31,−20}	{−29,−16}
Move down	{−38,−27}	{125,144}	{138,155}	{135,147}	{13,23}
Move close	{−64,−53}	{2,10}	{158,165}	{166,170}	{167,170}
Move far	{−2,6}	{62,71}	{133,144}	{145,163}	{161,170}

**Table 2:** Each intention’s prediction accuracy using different approaches.

Algorithm	Our Method	SVM	LDA	KNN
Give the big red cylinder	99%	92%	38%	92%
Give the middle black cylinder	100%	100%	52%	96%
Give the small blue cylinder	98%	81%	84%	93%
Need the big red cylinder	100%	100%	76%	100%
Need the middle black cylinder	100%	100%	66%	100%
Need the small blue cylinder	100%	99%	82%	100%
Rotate arm	100%	100%	100%	100%
Rotate hand	100%	100%	99%	100%
Move up	98%	100%	100%	100%
Move down	100%	99%	100%	100%
Move close	100%	100%	100%	100%
Move far	100%	100%	98%	100%

## Data Availability

The data that support the findings of this study are available from the corresponding author upon reasonable request.
